# Towards Efficient Building Designing: Heating and Cooling Load Prediction via Multi-Output Model

**DOI:** 10.3390/s20226419

**Published:** 2020-11-10

**Authors:** Muhammad Sajjad, Samee Ullah Khan, Noman Khan, Ijaz Ul Haq, Amin Ullah, Mi Young Lee, Sung Wook Baik

**Affiliations:** 1 Digital Image Processing Laboratory, Islamia College Peshawar, Peshawar 25120, Pakistan; Muhammad.sajjad@icp.edu.pk; 2 Sejong University, Seoul 143-747, Korea; samee@sju.ac.kr (S.U.K.); noman0869@sju.ac.kr (N.K.); ijazulhaq@sju.ac.kr (I.U.H.); qamin3797@gmail.com (A.U.); miylee@sejong.ac.kr (M.Y.L.)

**Keywords:** cooling load, energy consumption, energy efficient building, GRU, heating load

## Abstract

In the current technological era, energy-efficient buildings have a significant research body due to increasing concerns about energy consumption and its environmental impact. Designing an appropriate energy-efficient building depends on its layout, such as relative compactness, overall area, height, orientation, and distribution of the glazing area. These factors directly influence the cooling load (CL) and heating load (HL) of residential buildings. An accurate prediction of these load facilitates a better management of energy consumption and enhances the living standards of inhabitants. Most of the traditional machine learning (ML)-based approaches are designed for single-output (SO) prediction, which is a tedious task due to separate training processes for each output with low performance. In addition, these approaches have a high level of nonlinearity between input and output, which need more enhancement in terms of robustness, predictability, and generalization. To tackle these issues, we propose a novel framework based on gated recurrent unit (GRU) that reliably predicts the CL and HL concurrently. To the best of our knowledge, we are the first to propose a multi-output (MO) sequential learning model followed by utility preprocessing under the umbrella of a unified framework. A comprehensive set of ablation studies on ML and deep learning (DL) techniques is done over an energy efficiency dataset, where the proposed model reveals an incredible performance as compared to other existing models.

## 1. Introduction to Residential Building Energy

**Figure 1:**
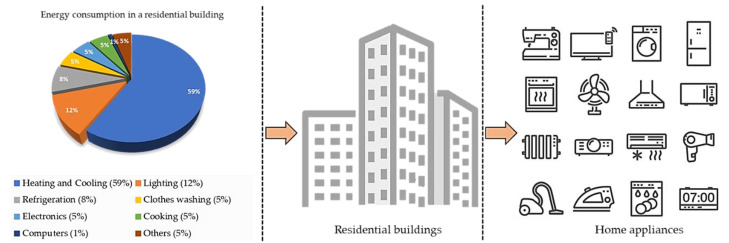
Massive amount of energy is consumed in the residential sector because various electrical appliances are installed.

Due to the rapid growth in economy and population during last few decades, the consumption of electrical energy has been rapidly increasing day by day [1]. In 2018, the International Energy Agency (IEA) reported that most of the electrical energy is spent in residential buildings, and the demand for energy is rising every year due to excess usage of energy appliances as shown in Figure 1. Existing studies reveal that residential buildings consumed more energy in summer and winter seasons, which totally depends on the building architecture and occupied area [2].

Internal and external environment temperatures also have an effect on the total energy consumption in a building [3]. Therefore, precise prediction of HL and CL is important in order to provide a luxurious life for occupants [4,5]. HL is described as the total amount of required heat energy to keep the room temperature normal, while CL is the sum of thermal energy necessary to be eliminated from a cooling area in order to keep the temperature at an appropriate level [2].

Before evaluating the thermal load, it is important to know the infrastructure of buildings, because energy consumption is reliant on their physical attributes. Basically, four tools are employed to predict the CL and HL of buildings: simulation modeling, engineering calculations, statistical models, and ML models [6]. The simulation model is commonly used to simulate energy efficiency based on prior information, but it is a very difficult and time-consuming model because it requires more skill to operate. For instance, Bagheri et al. [7] considered the simulation methods in terms of its applications and limitations in the domain of energy performance. The second tool utilized complicated mathematical formulas according to its principles to efficiently predict energy load. Next, a statistical tool is used to evaluate linear regression models for residential energy consumption prediction, and later, the performance of the model is enhanced by modifying different parameters. The final tool is ML, which is a subset of statistical techniques, but it has the potential to learn from real data and predict the desired outputs. Further, it assists civil engineers in evaluating the ingredients used in the building design. For instance, support vector regression (SVR), clustering, and Gaussian-based regression are active ML approaches in energy predictions [6].

ML algorithms can be broadly categorized into two main groups (i.e., supervised and unsupervised) based on diverse learning style. The predicted output variables are available in the case of supervised learning, while the unique labeled output does not occur in an unsupervised learning strategy. The current study focuses on a supervised learning approach because an energy efficiency dataset has labeled data. Artificial neural networks (ANNs) have gained attention among supervised learning techniques due to nonlinear relationships within the data. Moreover, the activation function of ANN can predicted the desired outputs, which indicate the nonlinearity with various input attributes [6]. Numerous ANN architectures, including recurrent networks, radial basis function, and feedforward, are used for energy prediction. Besides ANN, researchers have mostly implemented the multilayer perceptron (MLP) model, where information flows in a single direction with multiple layers. The MLP model comprises three basic layers (input, hidden, output) consisting of neurons with weighted functions. In case of complex data processing, the existing model is altered by increasing the number of neurons and hidden layers.

Managing huge and complicated energy consumption data is formidable for ANN, while researchers have criticized this network due to low transparency in the model [8]. Sensitivity analysis (SA) is broadly applied to analyze the relationship between variables. For precise energy forecasting, ANN shows better performance if irrelevant inputs are removed [9]. Sensitivity analysis about the mean (SAAM) is one of the conventional strategies, where changes of dependent variables are recorded while independent variables are kept in a specified range by computing the mean [10]. The key benefits of SAAM are simple interpretation, easy implementation, and application, along with statistical analysis [11]. In addition, state-based sensitivity analysis is a global SA method in which separate variables are varied independently and the rest of the variables are changed concurrently to obtain the reliant attributes [8].

In residential buildings, there are various factors that influence energy consumption, such as consumer’s behavior and building architecture. Therefore, building-structure-related data play a key role in developing an efficient energy model. Moreover, the height of buildings, construction materials, and areas such as wall, roof, and glazing are the main attributes in the current research. The simulated method in [12] performs a pivotal part in improving building constructions, and it can also accurately depict real assessments of different building designs to predict HL and CL [13]. On the other hand, most of the researchers get full advantages by applying DL models on different domains, such as movie and video summarization [14,15], energy forecasting [16], biological data analysis [17], violence detection [18,19], and action recognition [20]. In this study, we explored numerous ML and DL models for the prediction of HL and CL using an energy efficiency dataset. The potential of sequential models for this dataset has not been thoroughly explored till date. Therefore, GRU has an optimal preference to predict HL and CL as there exists an intensely independent relationship between data. We conduct two types of experiments. First, we enhance the existing performance in which HL and CL are predicted separately. Second, a multi-output prediction is performed through the same architecture. The relevance of this work can enable engineers to solve major structural issues when designing an energy-efficient building.

There is no existing work that utilized GRU for this dataset till date. Therefore, in the current study, we utilize the sequence learning model GRU for non-sequential data by examining various parameters. The second limitation is the unavailability of preprocessing methods, including polynomial and min–max normalization for HL and CL. In this study, the simulation data first pass through a preprocessing step where outliers are removed, scattered data is normalized in a specific range, and increase the number of features. Next, the refined data are fed into the GRU network to extract silent hidden patterns. Finally, we evaluate the error in different metrics, such as mean absolute error (MAE), relative mean absolute error (rMAE), mean square error (MSE), relative mean square error (rMSE), root mean square error (RMSE), relative root mean square error (rRMSE), mean average percentage error (MAPE), and relative mean average percentage error (rMAPE). The major contributions of this study are summarized below:

- It is a common fact that the performance of a deep model is directly depends on the input data. In this study, energy efficiency dataset is used that contains a limited number of attributes with values in a different range, which cause overfitting and take extra time to converge. To address these issues, first, we pass the input data through a preprocessing layer where the number of features increased using a polynomial equation and min–max normalization process is applied to remove outliers and normalize the data in a particular range.
- Existing models in the literature are trained separately for HL and CL prediction, which requires a tedious and time-consuming job. In contrast, the proposed framework has a generalized ability in which the same architecture can be used for both SO and MO that predict HL and CL concurrently.
- DL models always reveal a convincing performance compared with traditional ML models. Therefore, we propose a sequence learning model GRU, which learns discriminative features and efficiently predicts the HL and CL. We also conduct a comparative study between ML and DL techniques to show the superiority of DL models.
- We verify experimentally that the proposed framework outperforms state-of-the-art techniques using the hold-out and 10-fold methods. To check the effectiveness of the proposed framework, we evaluateit on various metrics, such as MAE, rMAE, MSE, rMSE, RMSE, and rRMSE.

The rest of the paper is categorized into four main sections. Section 2 briefly discusses the literature study about HL and CL prediction. Section 3 explains the proposed methodology, followed by comprehensive experiments in Section 4. Section 5 concludes this study with future research direction.

## 2. Literature Review of HL and CL Prediction

The literature study for HL and CL prediction in buildings is mainly divided into four major classes: residential, educational, commercial, and mixed. According to statistics in [21], 30% of the literature is based on residential building energy. Through Ecotect software, Tsanas and Xifara [12] simulated 12 distinct building structures to predict HL and CL. After considering all the various permutations of input variables, 768 building designs were generated. During the simulation of building designs, heating, ventilation, and air-conditioning HVAC rules were pursued. Through numerous ML techniques, various researchers analyze these data for precise prediction. Based on the prominent contribution of Tsanas and Xifara [12], the existing literature is summarized in Table 1. Although the dataset has been prepared via a simulated tool, but there is lack of data related to building infrastructure and materials. The dataset used in this study is publicly accessible and extensively used for research study by exploring its applications related to energy. Simulated data play a significant role when designing the architectures of a building. The terms used in the existing studies are listed in Table 1.

Tsanas and Xifara [12] conducted a detailed statistical study of density and scatterplots. The performance outcomes of the statistical analysis approach are mainly used for nonlinear problems. From Table 1, it can be observed that few studies have applied ANN on energy efficiency dataset [24,32], although others follow the ensemble strategy by integrating different methods [22,25,29,31]. To the best of our knowledge, only one article exists that applied a deep neural network (DNN) to predict HL and CL, presented by Sekha et al. [4]. The efficiency of DNN is better as compared with other traditional algorithms, such as Minimax Probability Machine Regression (MPMR) and Gaussian Process Regression (GPR). Moreover, the traditional approaches did not mention the model parameters, such as processing elements, activation functions, and numbers of layers. To achieve a remarkable performance on any models, analysis of data is essential to identify the significant and insignificant inputs. In this regard, Roy et al. [2] proposed a nonparametric regression model known as Multivariate Adaptive Regression Splines (MARS) that splits the data and fit each interval into a basis function. Principal component analysis (PCA) is also applied for ideal features selection and dimensionality reduction, which eradicates the multilinearity problem. Nilashi et al. [31] reported that PCA targets four main aspects: retrieving essential information, reducing the dimension of data, simplifying the information, and analyzing architecture-related observations. Most of the articles did not utilize the SA approach for the prediction of HL and CL as shown in Table 1.

The techniques for quantitative SA are classified into local and global [37]. For instance, the input instances were impartial with each other; therefore, Ardjmand et al. [8] defined a regression-based strategy in which conventional SA techniques, such as sampling, regression-based, and variance-based, are expanded to state-based sensitivity analysis (SBSA). This means that modifying one variable value would influence the others; therefore, it is not realistic for fixed values for certain inputs in local SAs. On the other hand, the global SA adjusts the ideal input value, while in the case of multidimensionality, it takes the average number of variable inputs [38].

The efficiency of a mathematical model is also influenced by many assumptions in order to predict energy HL and CL separately. In majority of the works, the HL and CL are predicted in an SO fashion; however, we develop such a model that can be utilized for both SO and MO. Another primary consideration for enhancing the efficiency of a predictive model is preprocessing of data. Therefore, Kumar et al. [35] followed the ensemble technique with a proper attribute selection and preprocessing method to efficiently predict energy in real time. To boost the model efficiency, it is necessary to pass the data through the preprocessing stage. Notably, MSE, MAE, RMSE, and MAPE were common evaluation metrics used by researchers for model assessment, but in this research, we also use extra metrics for evaluation, including rMAE, rMSE, rRMSE, rMAPE.

## 3. Methodology for HL and CL Prediction

**Figure 2:**
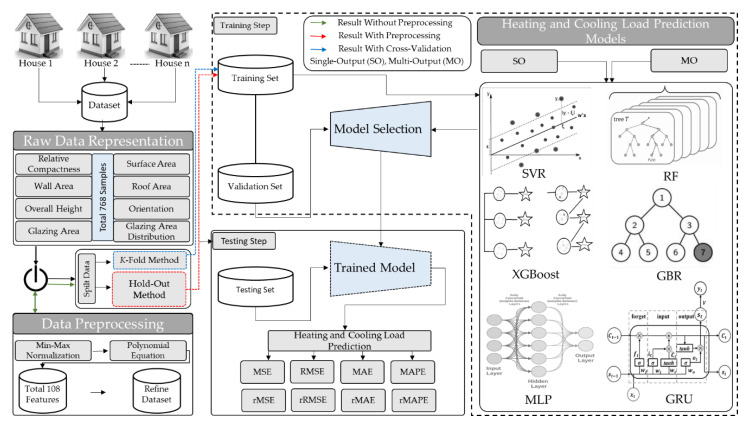
The proposed framework for precise prediction of HL and CL through energy efficiency data using sequential learning model.

The primary goal of the proposed framework is to efficiently predict the HL and CL that will assist engineers in building energy-efficient buildings. A comprehensive set of experiments are conducted on ML and DL using hold-out and cross-validation methods. The proposed framework is mainly categorized into three steps, as shown in Figure 2. In the first step, we perform the preprocessing on raw data related to the building. In the second step, the polished data are redirected to GRU, where they learn hidden patterns in both forward and backward fashion. In the third step, the softmax layer generates the desired output and evaluates the performance of the model through various evaluation metrics. All the acronyms used throughout the paper are defined in Table 2.

### 3.1. Data Acquisition and Preprocessing

**Figure 3:**
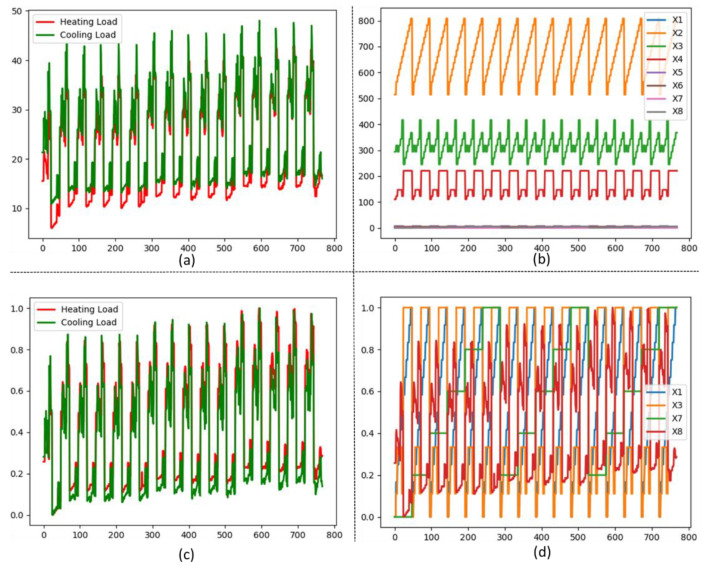
(**a**) Visual representation of actual HL and CL data where *x*-axis shows the number of samples and *y*-axis illustrates the range of samples; (**b**) overall attributes in the dataset; (**c**) normalized sample value of HL and CL; (**d**) normalized value of 4 attributes.

Existing simulated data comprise various attributes related to building structure, such as relative compactness, overall area, height, orientation, and distribution of the glazing area. The performance of deep models depends on the characteristics of the data for training. For instance, if the input data are well organized, then they assist in efficient performance. In this study, we employ two techniques in the preprocessing step to refine the simulation because each of the attribute data in the dataset is scattered with each other that trigger the outlier issue. Therefore, we apply min–max normalization to remove outliers and arrange all the values in the range of 0 and 1. The alteration effect on data before and after normalization is visualized in Figure 3. Similarly, samples of the dataset are very few, and prior researchers always tried to tune a model for enhancing the performance of the model over limited data. From various computer vision problems, it has been proved that the DL model depicts a remarkable performance on a massive number of data [39]. However, in our case the total samples in the dataset are fewer; therefore, we apply a polynomial equation to increase the number of features. In this perspective, we also check the generalized capability of the model and decrease the overfitting probabilities during training. In addition, such model can be applicable for new data predictions in the future.

Before applying the polynomial equation, first, we made four sets of couples from the given eight attributes, where each couple is expanding up to the sixth power with various possible combinations of pairs. Equation (1) presents a polynomial process applied to a single pair of attributes.

(1) $$Fv=(x,\;y,\;x^{2},xy,\;y^{2},\;x^{3}\ldots \ldots \ldots xy^{5},\;y^{6}).$$

where *Fv* represents the polynomial feature vector and *x*, *y* denotes two different attributes, where from each pair, 27 diverse feature vectors are produced, while in our case, we have 8 pairs, so the total possible feature vectors become (27 × 8 = 108), which is our desired output. 

### 3.2. Multi-Output (MO) Regression Model

Various traditional approaches have been developed to efficiently predict HL and CL in an SO manner, where a model is trained two times for each prediction. The energy efficiency dataset contains a total of eight features (relative compactness, surface area, wall area, roof area, overall height, orientation, glazing area, and glazing area distribution) and two labels (HL and CL). Hence, there are no separate attributes and samples for HL and CL prediction; therefore, these models were first utilized for CL data prediction and then trained again for HL, which is a laborious and headache job.

**Figure 4:**
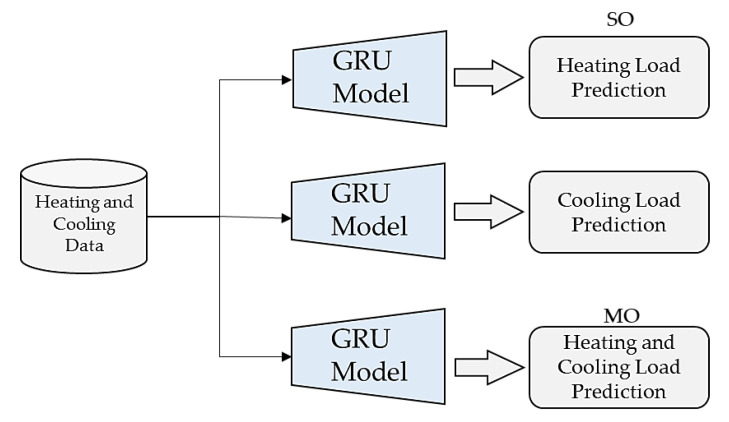
Difference between SO and MO in the prediction of HL and CL using the GRU model.

In this paper, we propose an efficient model for both SO and MO with static parameters. First, we improve the performance of the model in SO, and then extra experiments are performed to evaluate the error rates of HL and CL in an MO fashion to save time. To the best of our knowledge, there is no existing model that can generate MO through a sequential learning approach. We performed comprehensive experiments on both SO and MO using various ML and DL techniques to find the optimal model that predicts the HL and CL in an efficient way (Figure 4).

### 3.3. Machine Learning (ML)

#### 3.3.1. Support Vector Regression (SVR)

Support vector machine (SVM) is a supervised ML approach mainly used for classification and regression problems. It has been proved from earlier studies that SVM shows incredibly better performance than other supervised learning algorithms because its formulation is based on structural risk minimization instead of empirical. In this paper, we deal with CL and HL prediction as a regression problem; therefore, we use SVR. The main goal of SVR is convex means that global optimum is always converge in this approach, first, the input data *x* is mapped via nonlinear mapping into *n*-dimensional feature space. In the consequent feature space, we build a linear model for the prediction of CL and HL. The mathematical background of the linear model is presented in Equation (2).

(2) $$M_{\left(x,ա\right)}=\;\left(\sum_{i=1}^{n}{ա}_{i\;}h_{i\;}\left(x\right)+\;b\right).$$

where nonlinear transformations are denoted by *h* (*i*) (*x*), *i* = (1, …... *n*) as most of the time the bias “*b*” term is considered zero; therefore, it is ignored. The SVR model is totally dependent on the training data; therefore, it always tries to minimize the error rate and model complexity by reducing *║ա║*^2^, which is the main objective.

(3) $$\left(ɱ\mathrm{in}1/2{║ա║}^{2}+C\overset{m}{\underset{j=1}{\sum }}\xi_{j}+\;\xi_{j}^{*}\right)$$

$$\mathrm{Such}\;\mathrm{that}\;Z_{j}-M_{\left(x_{j},ա\right)}\leq \varepsilon +\xi_{j}^{*}$$

$$M_{\left(x_{i},ա\right)}-z_{i}\leq \varepsilon +\;\xi_{j}.$$

$$\xi_{j},\;\xi_{j}^{*}\;\geq \;0,\;j=\;(1,\;.\text{…},\;m).$$

In Equation (3), *ε* denotes the loss function, while *ξ_j_* and $\xi_{j}^{*}$ are the slack non-negative variables that determine the training sample deviation. The parameter *C* > 0 is used to measure the fitting of the samples, and *ա* illustrates the regression weights of the SVR model. Generally, the optimization task can be easily handled if converted into a dual problem by applying the dualization and Lagrange multipliers techniques. These methods are mathematically elaborated in Equation (4).

(4) $$L\;=\;\left(\begin{array}{c} \frac{1}{2}{║ա║}^{2}+C\sum_{j=1}^{m}(\xi_{j}+\;\xi_{j}^{*})-\;\sum_{j=1\;}^{m}\sigma_{j}^{*}\;(\varepsilon +\;\xi_{j}^{*}\;-Z_{j}+\;M_{\left(x_{j},ա\right)})-\\ \sum_{j=1\;}^{m}\sigma_{j}\;(\varepsilon +\;\xi^{*}\;-Z_{j}+\;M_{\left(x_{j},ա\right)})-\;\sum_{j=1\;}^{m}\;(\lambda_{j}\varepsilon +\;\lambda_{j}^{*}\xi_{j}^{*}\;)\;, \end{array}\right)$$

In the Lagrange *L* equation, *σ_j_*, $\sigma_{j}^{*}$, *λ_j_*, $\lambda_{j}^{*}\;$≥ 0 refer to Lagrange multipliers. According to the consideration saddle point situation, the partial derivatives of the Lagrange variable (*ա*, *b*, *ξ_j_*, $\xi_{j}^{*}$) will disappear during optimality. Finally, in Equation (5) is the dual optimization along with the preceding steps.

(5) $$g\left(x\right)\;=\;\sum_{j=1\;}^{Nsv}\;\left(\sigma_{j}-\sigma_{j}^{*}\right)K(x_{j}-\;x\;)$$

In the dual optimization procedure, *K* depicts the number of vectors in feature space and kernel function. The dot product of two vectors with kernel is represented in Equation (6).

(6) $$K\left(x,x_{j}\right)\;=\;\sum_{j=1\;}^{n}\;g_{j}\left(x\right)\;g_{j}\left(x_{j}\right)$$

In this study, we use a Gaussian radial basis function (RBF) with its kernel parameters *Ύ'* in order to manage the nonlinearities between the input data and their perceptive class.

#### 3.3.2. Random Forest (RF)

Random forest (RF) is a supervised learning algorithm and ensemble approach of randomized decision-making trees (DMTs). A DMT is a non-parametric ML algorithm that establishes a model in the form of a tree structure and divides the given records into smaller chunks until only one record remains in the subgroup. The final and internal sets are referred to as leaf and root nodes, respectively. The particular DMT is utilized for an unstable system that is totally dependent on data, so limited data affect the entire structure. To tackle this problem, a collection of DMTs is utilized to select target values based on average predicted values for all individual trees. Typically, RF follows the bags and boosts strategy in which they integrate different models sharing common information in order to produce several individual trees [40]. Multiple hyper parameters are required to tune the RF, but the number key parameter is the number of independent trees in the forest. To find an efficient model in terms of accuracy and time complexity, it is necessary to tune the model on different parameters.

#### 3.3.3. XGBoost

XGBoost is a supervised learning approach that is applicable to both regression and classification problems. Here, we use multiple features *X_i_* for training data to predict the target variable *Y_i_*. XGBoost stands for “Extreme Gradient Boosting”, which follows an ensemble learning strategy, including regression tree and classification mechanism. Let us assume {*T*_1_……. *T_n_* (*x_i_*, *y_i_*) where, *i* = 1, …, *n*}, where *x_i_* and *y_i_* represent the training samples and the appropriate class labels. To get the final predicted score, all the individual scores are combined via additive function *A* as depicted in Equation (7).

(7) $$y_{i}^{*}\;=\;\sum_{a=1\;}^{A}\;f_{a}\left(X_{i}\right),\;f_{a}\varepsilon \;F.$$

where *f_a_* is the number of gradient boosting trees and functional spaces for all trees. The two additional functions (i.e., training and regularization term) are illustrated in Equation (8):

(8) $$O_{\theta }\;=\;\sum_{i\;}^{m}\;1\left(y_{i},y_{i}^{*}\;\right)+\sum_{a=1\;}^{A}\;ѱ\left(\;f_{a}\right).\;$$

where the measurement of the loss function between target and predicted class is illustrated as 1 and *ѱ* is represent the regularization term used for handling the overfitting issue.

In XGBoost for each level ‘*t*’, the additive training approach is followed for the prediction of each class label $y_{i}^{*}$. Mathematically, it can be shown in Equation (9).

(9) $$y_{i}^{*}{}^{t}\;=\;\sum_{a=1\;}^{A}\;f_{a}\left(x_{i}\;\right)=y_{i}^{*}{}^{t-1}\;+\;f_{t}\left(x_{i}\;\right).\;$$

After applying the tree boosting method, Equation (8) becomes

(10) $$O_{\theta }{}^{t}\;=\;\sum_{i\;}^{m}\;1\left(y_{i},y_{i}^{*}{}^{t-1}+\;f_{t}\left(x_{i}\;\right)\right)+ѱ\left(\;f_{t}\right).$$

Equation (10) is obtained by dividing the leaf node to get the final score after a series of evaluations.

### 3.4. Deep Learning (DL)

#### 3.4.1. Multilayer Perceptron (MLP)

**Figure 5:**
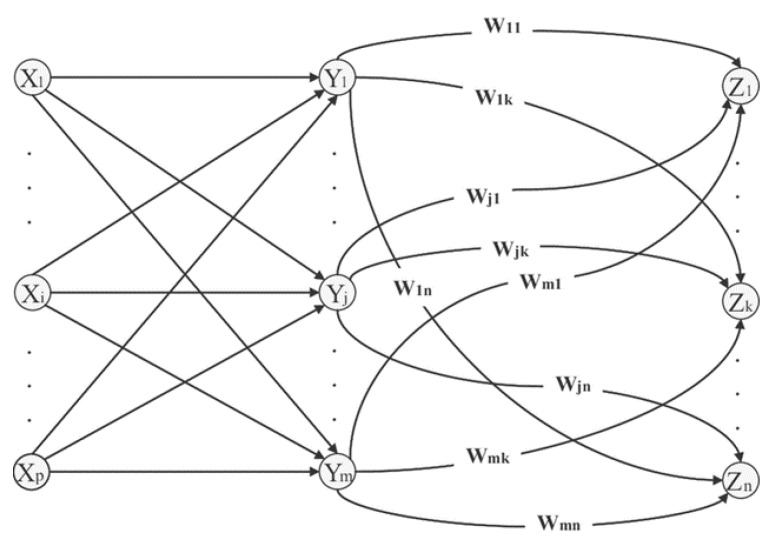
Backpropagation of MLP architecture with one hidden layer.

Inspired by the human brain, McCulloch and Pitts proposed the concept of ANN [41], which has been widely used in various research domains [42]. One of its fundamental potentials is generating the nonlinear relation map between the input and the output. In the current study, we choose MLP among various types of ANNs because it is one of the trustworthy methods designed for prediction problems. Our MLP architecture comprises three layers along with computational units called neurons, as shown in Figure 5. To achieve the actual and predicted outputs precisely, it is necessary to design an efficient model. Moreover, let us assume the input vectors are denoted by I; then the final output for the *n*-th neuron is formulated by Equation (11):

(11) $$O_{k}=\;A\left(\overset{j}{\underset{i=1}{\sum }}I_{i\;}W_{ik\;}+\;b_{k}\right).$$

where *I* illustrate the input samples, and *W*, *b*, and *A* represent the weights, bias, and activation function, respectively.

#### 3.4.2. Gated Recurrent Unit (GRU)

**Figure 6:**
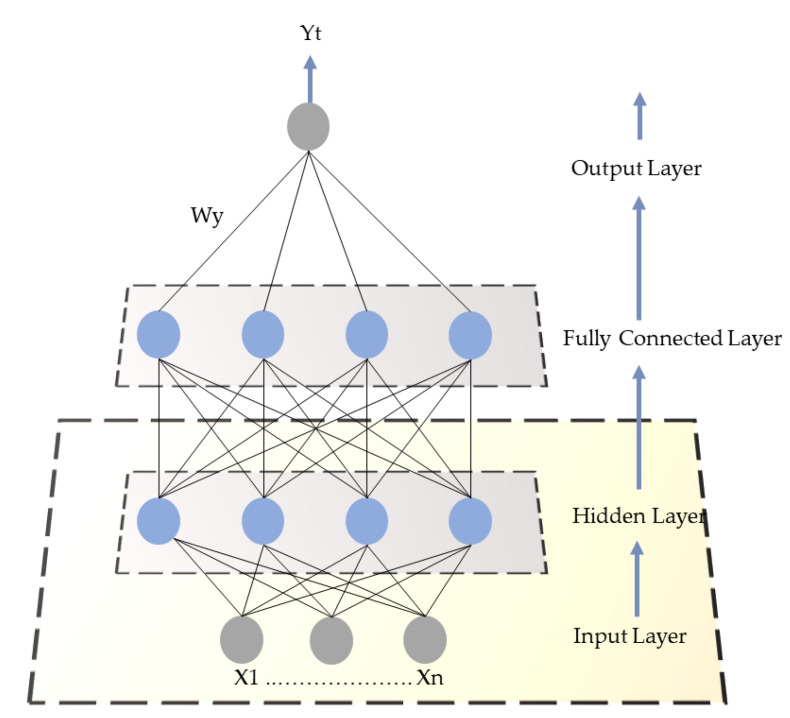
The GRU architecture for HL and CL prediction.

GRU is the most common sequence learning model of Recurrent Neural Network (RNN), which is basically used to overcome the vanishing gradient issue [43]. GRU contains two main gates (i.e., update and reset gate), that determine which information is to be transferred to the output layer as shown in Figure 6. The unique capability of these two gates is that they can store information for the long term and they do not remove irrelevant information related to the prediction. They can be considered as a subset of Long Shot-Term Memory (LSTM) due to similarity in architecture and performance. In the current study, we use GRU because it gives a remarkable performance on smaller numbers of sample data as well as on more samples. Furthermore, it trains faster due to a smaller number of parameters. The mathematics behind this network is shown in Equations (12)–(15).

*Z_t_* = *Θ*(*W*_(*z*)_*X_t_* + *U*_(*z*)_*h*_(*t*−1)_). (12)

In the update gate, as the input *X_t_* is tied to the network node, it is multiplied by its weights *W*_(*z*)_. Similarly, *h*_(*t*−1)_ retains the information of the earlier cell units and is multiplied by its particular weights *U*_(*z*)_. The outcomes of both are integrated and flatten the result between the range of 0 and 1 through sigmoid activation function.

*r_t_* = *Θ*(*W*_(*r*)_*X*_*t*_ + *U*_(*r*)_*h*_(*t*−1)_). (13)

The reset gate is almost similar to the update gate, but the difference is only in weights and functionality. This gate is basically used to decide how much previous information is to be forgotten. As *h*_(*t*−1)_ and *X_t_* are multiplied with their subsequent weights. After that, the sigmoid function is applied to the merged results.

(14) $${ĥ}_{t}=\;\tan h\left(\;Wx_{t}\;+\;r_{t}\;•\;Uh_{t-1}\right).$$

The reset of the gate memory content is used to hold the relevant pattern information from the earlier cell gate. Here, two operations are performed: first, the input sequence *X_t_* is multiplied with its corresponding weights *W*, and second, an element-wise operation is performed between *r_t_* and *Uh*_(*t*−1)_, which will take a decision in removing the information from the earlier time steps. Furthermore, it sums up both the generated output and the employed nonlinear tan*h* activation function.

(15) $$h_{t}=\;\left(z_{t}\;•\;h_{t-1}+\;(1-z_{t})\;•\;{ĥ}_{t}\right).$$

Finally, the network calculates the *h_t_* vector, and the last memory of the current time step holds the current unit information and forwards it to the next layer of the network in order to update the gate. Furthermore, it contains information about the current and previous steps. In this unit cell, three basis operations are performed, first, to update the gate element-wise product operated between *z_t_* and *h*_(*t*−1)_. Second, again element-by-element multiplication is done between (1 − *zt*) and *ĥt*. In the last operation, both operations 1 and 2 are incorporated, and the final output is produced.

## 4. Experimental Results

### 4.1. System Configuration

We substantiate the effectiveness of the proposed GRU model using an energy efficiency dataset that is publicly available on the University of California Irvine (UCI) repository. The model is trained over TITAN X (Pascal)/PCLe/SSE2 GPU with Intel Core i5-6600 processor, 64 GB RAM, and Windows 10 operating system. The implementation is performed in Python with Keras DL framework with TensorFlow at the backend and Adam optimizer with 100 epochs, 0.0001 learning rate, 0.9 momentum, 16 batch size, and 20 units in GRU. Two types of experiments are conducted, including hold-out and cross-validation on the energy efficiency dataset, which are further categorized into SO and MO with and without preprocessing. Furthermore, in the hold-out method, the data are divided into training set 80% and testing 20%, and from the training set, we select 10% data as a validation set. On the other hand, in the cross-validation process, the entire data are divided into 10 equal parts. After that, one chunk is used for the testing set, and the remaining ten chunks are considered as training set. This process is repeated until each chunk is tested.

### 4.2. Dataset Description

The dataset used for this study is proposed by [12] with no additional characteristics. Through an elementary cubes process, 12 residential building shapes were simulated, each comprising 18 elements, and the total volume of the buildings was 771.75 m^3^. The building relative compactness is computed by evaluating the total areas of the building structure and position structure when the total volume of the building is equal to the position structure. The selection was made by the newest and most common materials used during constructing building industry and by the lowest U-value. Building structure features and their related (U-values appear in parenthesis): walls (1.780), floors (0.860), roofs (0.500), and windows (2.260). The simulated data is assumes that the actual buildings are located in Athens, Greece. The interior layout was set as clothing: 0.6 clo; room temperature: 21 °C; thermal insulation that is suitable for a particular weather condition; humidity rate: 60%; air velocity: 0.3 m/s; and a total of 300 lux lights. The heating properties have shown 95% efficiency with a thermostat range of 19–24 °C that is working 15–20 h on weekdays and 10–20 h on weekends. Three different percentages were utilized for the glazing area of a building: 10%, 25%, and 40%. Furthermore, the glazing area was categorized into five various scenarios: uniform, 25%; north, 55%; east, 55%; south, 55%; and west, 55%. In addition, four directions were indicated by 2, 3, 4, and 5, which represent north facing, south facing, east facing, and west facing, respectively.

The energy efficiency dataset contains 12 shapes of a building along with 3 glazing areas, 5 different distribution scenarios for each glazing areas, and 4 directions, which are associated with 720 samples. However, if we enter the structure of the 12 buildings, which have no glazing area along 4 orientations, then the total becomes 768 buildings with respective values of HL and CL. The key characteristics of inputs and outputs are given in Table 3.

### 4.3. Evaluation Metrics

To assess the variation between the actual and predicted heating and cooling load, it is necessary to evaluate the effectiveness of the regression model via various evaluation metrics, such as MSE, RMSE, MAE, and MAPE. Basically, MSE calculates the average square value of the difference between the target and predicted values via the regression model. RMSE is commonly used for a regression problem, which is the root squared difference between the actual and predicted values, and MAE is the linear score in which the individual weighted differences are considered equally. Finally, the MAPE metric computes the prediction accuracy in percentage. The mathematical representation of all these metrics is depicted in Equations (16)–(19).

(16) $$\mathrm{MSE}=\frac{1}{n}\sum_{1}^{n}{\left(y-\hat{y}\right)}^{2}$$

(17) $$\mathrm{MAE}=\;\frac{1}{n}\sum_{1}^{n}\left|y-\hat{y}\right|$$

(18) $$\mathrm{RMSE}=\sqrt{\frac{1}{n}\sum_{1}^{n}{\left(y-\hat{y}\right)}^{2}}$$

(19) $$\mathrm{MAPE}=\;\frac{100\%}{n}\sum_{1}^{n}\frac{\left|y-\hat{y}\right|}{y}\;$$

### 4.4. Performance of ML and DL Methods for SO

**Figure 7:**
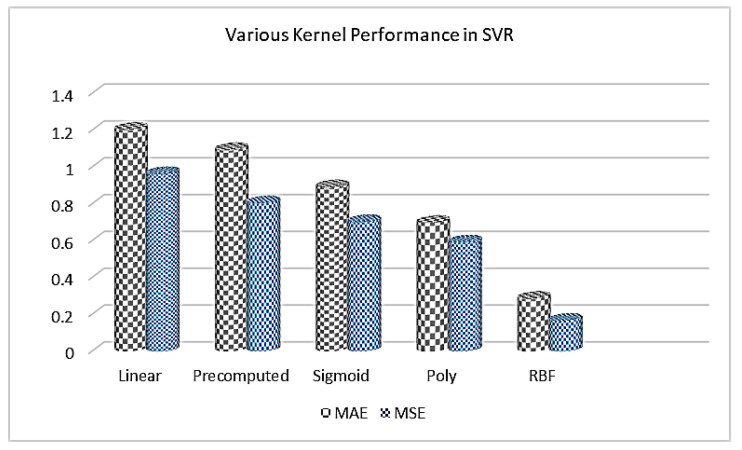
Numerous kernel performances of SVR in the prediction of HL and CL.

In this study, we performed various experiments on ML and DL methods to select the optimal model for SO. The experiments were performed with and without preprocessing followed by two strategies: hold-out and cross-validation. In the ML models, XGBoost beat all other ML algorithms in both cases because it utilized the ensemble learning strategy. In HL and CL prediction, it showed remarkable performance as depicted in Table 4. Further, we checked the prediction error in various kernels in SVR as shown in Figure 7. 

In contrast to ML algorithms, GRU significantly predicted the HL and CL in both processed and unprocessed data in DL algorithms. In refined data, GRU achieved 0.0102, 0.0003, 0.0166, and 0.0284, for MAE, MSE, RMSE, and MAPE in HL prediction and 0.0167, 0.0006, 0.0247, and 0.0368 for MAE, MSE, RMSE, and MAPE in CL prediction, respectively. The rest of the algorithm’s performance are illustrated in Table 4**.**

It is clear from Table 4 that our preprocessing strategy significantly reduced the error rate in all methods. Besides hold-out, we also did the cross-validation for the prediction of HL and CL, where again XGBoost showed better performance than other ML methods, while GRU dominated all methods, including XGBoost and MLP, because it learns more from data in an efficient way. The comprehensive experiments are shown in Table 5.

### 4.5. Performance of ML and DL Methods for MO

In this section, we conducted numerous experiments on ML and DL models in order to pick the optimal model on the basis of their performance. State-of-the-art models generated output in a SO fashion that required more time for the prediction of HL and CL. There is no existing work that employed a sequence learning model to generate the desired output in MO style. The same set of experiments for hold-out and 10-fold cross-validation with and without refining data are conducted for MO model assessment. In the ML category, XGBoost showed a convincing performance, while the proposed model (GRU) showed overall dominancy in both hold-out and 10-fold cross-validation because it only keeps and learns the most prominent information to make HL and CL predictions and eliminates irrelevant information. Detailed results are given in Table 6.

**Figure 8:**
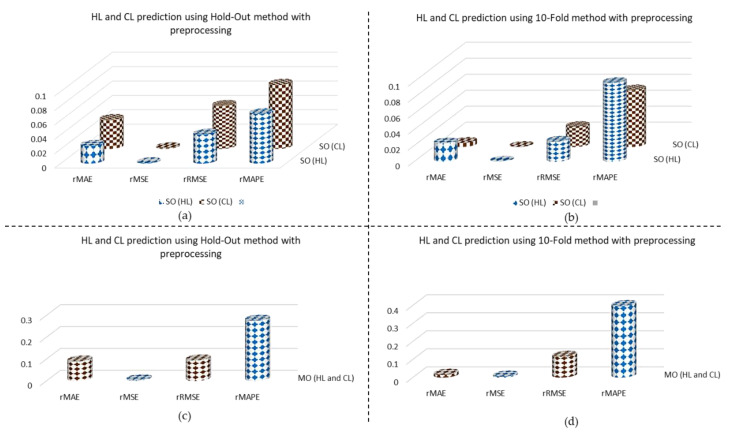
Experimental results of the proposed model (GRU) for SO and MO prediction using the hold-out and 10-fold methods.

The proposed model (GRU) is further evaluated through some extra metrics mostly used in statistical data analysis. The relative metrics compute the ratio between actual and error values. The graphical representation of various experiments in hold-out and 10-fold cross-validation are visualized in Figure 8.

**Figure 9:**
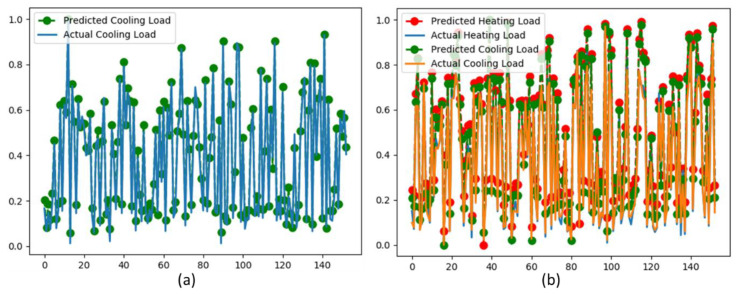
Visualization of prediction results obtained via the proposed model (GRU), where *x*-axis indicates the number of samples while *y*-axis represents the actual and predicted load; (**a**) the actual and predicted outputs of CL using the SO strategy; (**b**) the actual and predicted outputs of HL and CL using the MO strategy.

For more satisfaction of the proposed model, we also visualized actual and predicted load on both SO and MO as illustrated in Figure 9.

### 4.6. Comparison with State-Of-The-Art Models

In this section, we compared the results achieved through the proposed model (GRU) with existing approaches over energy efficiency dataset. The proposed model showed a remarkable performance on both hold-out and 10-fold cross-validation as compared to state-of-the-art models. Most of the researchers developed ML and ANN methods to evaluate HL and CL without utilizing a preprocessing technique, which sometimes generated false prediction. Furthermore, they repeated the training process to obtain the HL and CL values individually, which is a very tedious and time consuming job. In a such way, most of time the performance of the HL improved, but, the accuracy of the CL prediction decrease. In contrast, our proposed model is better than the others existing baseline models because, we employed a sequential learning model for nonsequential data which improved the SO and MO performances on both hold-out and 10-fold. Table 7 presents the SO results based on the hold-out technique with recent state-of the-art models [2,4,12,22,23,25,26,28,31,32,33,34,35,36,44]. For HL prediction, the proposed model (GRU) achieved the least error rates for MAE (0.0102), MSE (0.0003), and RMSE (0.0166). Similarly, the proposed model (GRU) achieved an incredible performance and attained the best results for CL (i.e., 0.0167, 0.0006, and 0.0247 for MAE, MSE, and RMSE, respectively). 

## 5. Conclusions and Future Research Direction

In this study, we proposed an intelligent framework for HL and CL prediction via a sequential learning model (GRU). First, we applied min-max normalization and polynomial equation in order to remove outliers, normalize all the sample values in specific range, and increase the number of features, respectively. Next, we conducted comprehensive set of experiments over ML and DL methods using hold-out and 10-fold cross-validation to choose the most favorable model in terms of accuracy. Finally, to validate the performance of the proposed model we evaluated it on numerous metrics such as MAE, rMAE, MSE, rMSE, RMSE, rRMSE. In the future, we aim to utilize evolutionary algorithms on different datasets and improve the existing performance of the model by utilizing some advanced preprocessing strategies.

## Figures and Tables

**Table 1:** Existing approaches for the prediction of HL and CL using energy efficiency dataset.

Reference	Learning Strategy	Feature Selection	Evaluation Metrics
Tsanas and Xifara [12]	RF, iteratively reweighted last squares (IRLS)	Mutual information, Spearman rank correlation coefficient, and *p*-value	MSE, MAE, MRE
Chou and Bui [22]	Fusion method (SVR + ANN), RF, SVR, CART, GLR, CHAID	-	RMSE, MAE, MAPE, R, SI
Cheng and Cao [23]	Evolutionary multivariate adaptive regression splines (EMARS)	MARS	RMSE, MAPE, MAE, R^2^
Ahmed et al. [24]	ANN and *k*-means cluster	-	Silhouette score
Sonmez et al. [25]	KNN and ANN (ABC, GA)	-	MAE, standard deviation
Alam et al. [26]	ANN	ANOVA	RMSE
Fei et al. [27]	ANN	-	MSE
Regina and Capriles [28]	DT, MLP, RF, SVR	-	MAE, RMSE, MRE, R^2^
Naji et al. [29]	ANFIS	-	RMSE, R, R^2^
Naji et al. [30]	ELM	-	RMSE, R, R^2^
Nilashi et al. [31]	EM and ANFIS	PCA	MAE, MAPE, RMSE
Nwulu [32]	ANN	-	RMSE, RRSE, MAE, RAE, R^2^
Duarte et al. [33]	DT, MLP, RF, SVM	-	MAE, RMSE, MAPE, R^2^
Roy et al. [2]	Multivariate adaptive regression splines, ELM, a hybrid model of MARS and ELM	MARS	RMSE, MAPE, MAE, R^2^, WMAPE, Time
Kavaklioglu [34]	OLS, PLS	-	RMSE, R^2^,
Kumar et al. [35]	ELM, online sequential ELM, bidirectional ELM	-	MAE, RMSE
Al-Rakhami et al. [36]	Ensemble learning applying XGBoost	-	RMSE, R^2^, MAE, MAPE
Sekhar et al. [4]	DNN, GRP, MPMR	-	VAF, RAAE, RMAE, R^2^, MAPE, NS, RMSE, WMAPE

**Table 2:** Parameters and their abbreviations.

Acronyms	Description	Acronyms	Description
CL	Cooling load	MAE	Mean absolute error
HL	Heating load	RMSE	Root mean square error
GRU	Gated recurrent unit	SA	Sensitivity analysis
SVR	Support vector regression	SVM	Support vector machine
ANN	Artificial neural network	PCA	Principal component analysis
MLP	Multilayer perceptron	DNN	Deep neural network
ML	Machine learning	SO	Single-output
DL	Deep learning	MO	Multi-output
RF	Random forest	GPR	Gaussian process regression
MSE	Mean square error	GBR	Gradient boost regressor
SVM	Support vector machine	DMTs	Decision-making trees
rMSE	Relative mean square error	rRMSE	Relative root mean square error

**Table 3:** Detailed description of energy efficiency dataset.

Variable	Building Information	Attribute	Total Values	Data Type	Units
**Input**	Relative compactness	X_1_	12	Real	None
	Surface area	X_2_	12	Real	m^2^
	Wall area	X_3_	07	Real	m^2^
	Roof area	X_4_	04	Real	m^2^
	Overall height	X_5_	02	Real	M
	Orientation	X_6_	04	Integer	None
	Glazing area	X_7_	04	Real	None
	Glazing area distribution	X_8_	6	Integer	None
**Output**	Heating load	Y_1_	586	Real	kWh/m^2^
	Cooling load	Y_2_	636	Real	kWh/m^2^

**Table 4:** Experimental results of various ML and DL models for SO prediction using the hold-out method.

**Method**	**Hold-Out without Preprocessing**							
	**HL**				**CL**			
	**MAE**	**MSE**	**RMSE**	**MAPE**	**MAE**	**MSE**	**RMSE**	**MAPE**
SVR	1.9532	1.5241	1.2345	1.3913	2.2143	1.6241	1.2744	1.7471
RF	2.4310	1.8701	1.3675	1.6714	2.4197	1.9875	1.4097	1.9032
XGBoost	1.8236	1.4797	1.2164	1.5941	2.1027	1.5579	1.2481	1.6179
GBR	2.3142	1.6091	1.2685	1.6721	2.3471	1.7928	1.3389	1.8932
MLP	1.7613	0.9781	0.9889	1.1741	1.9897	1.0899	1.0439	1.4869
GRU	1.3691	0.7215	0.8494	0.9315	1.4027	0.9791	0.9894	1.0132
**Method**	**Hold-Out with Preprocessing**							
	**HL**				**CL**			
	**MAE**	**MSE**	**RMSE**	**MAPE**	**MAE**	**MSE**	**RMSE**	**MAPE**
SVR	0.2855	0.1658	0.4072	0.5833	0.5662	0.6851	0.8277	0.9428
RF	0.3225	0.1924	0.4386	0.5312	1.0212	2.3355	3.7084	3.8192
XGBoost	0.2130	0.0911	0.3018	0.4120	0.4167	0.3566	0.5971	0.6580
GBR	0.3048	0.1467	0.3830	0.5269	0.9311	0.5971	2.7084	2.8149
MLP	0.0853	0.0075	0.0867	0.0988	0.0838	0.0074	0.0858	0.0897
GRU	0.0102	0.0003	0.0166	0.0284	0.0167	0.0006	0.0247	0.0368

**Table 5:** Experimental results of various ML and DL models for SO prediction using the 10-fold method.

**Method**	**Cross-Validation without Preprocessing**							
	**HL**				**CL**			
	**MAE**	**MSE**	**RMSE**	**MAPE**	**MAE**	**MSE**	**RMSE**	**MAPE**
SVR	2.0978	1.6463	1.2830	1.4192	2.2089	1.7574	1.3256	1.5303
RF	2.5421	1.9943	1.4121	1.6971	2.6532	2.0215	1.4217	1.7082
XGBoost	1.9347	1.5998	1.2648	1.4023	2.0458	1.7110	1.3080	1.5134
GBR	2.4235	1.7497	1.3227	1.5932	2.5346	1.8608	1.3641	1.7043
MLP	1.8724	1.4996	1.2245	1.4932	1.9835	1.6107	1.2691	1.6043
GRU	1.4802	0.9871	0.9935	1.0210	1.5913	0.8920	0.9444	1.1031
**Method**	**Cross-Validation with Preprocessing**							
	**HL**				**CL**			
	**MAE**	**MSE**	**RMSE**	**MAPE**	**MAE**	**MSE**	**RMSE**	**MAPE**
SVR	0.1941	0.0431	0.2076	0.3712	0.1830	0.0320	0.1788	0.2823
RF	0.2916	0.0981	0.3132	0.4312	0.2805	0.0870	0.2949	0.5024
XGBoost	0.1813	0.0334	0.1827	0.2715	0.1701	0.0231	0.1519	0.2529
GBR	0.2712	0.0849	0.2913	0.3108	0.2601	0.0738	0.2716	0.3914
MLP	0.0191	0.0091	0.0953	0.1076	0.0189	0.0080	0.0894	0.1289
GRU	0.0092	0.0001	0.0100	0.0391	0.0021	0.0001	0.0100	0.0282

**Table 6:** Experimental results of various ML and DL models for MO prediction using the hold-out method.

**Method**	**Hold-Out with Preprocessing**				**Hold-Out without Preprocessing**			
	**HL and CL**				**HL and CL**			
	**MAE**	**MSE**	**RMSE**	**MAPE**	**MAE**	**MSE**	**RMSE**	**MAPE**
SVR	0.7831	0.5479	0.7402	0.8922	3.5347	2.3701	1.5395	2.6368
RF	0.9867	0.7863	0.8867	0.9647	3.9375	2.5561	1.5987	2.8059
XGBoost	0.5182	0.4841	0.6957	0.7328	3.2439	2.1253	1.4578	2.5278
GBR	0.6798	0.6531	0.8081	0.9781	3.7294	2.4321	1.5595	2.7053
MLP	0.0953	0.0189	0.1374	0.2579	2.9124	1.9760	1.4057	1.9979
GRU	0.0368	0.0015	0.0387	0.1134	1.7519	1.0217	1.0107	1.0901
	**Cross-Validation with Preprocessing**				**Cross-Validation without Preprocessing**			
SVR	0.6975	0.4043	0.6358	0.7098	3.4438	2.2903	1.5133	2.8186
RF	0.8790	0.6901	0.8307	0.9767	3.8466	2.4650	1.5700	3.0077
XGBoost	0.4791	0.3765	0.6135	0.7452	3.1529	2.0344	1.4263	2.7096
GBR	0.5170	0.5536	0.7440	0.8062	3.6385	2.3412	1.5300	2.9071
MLP	0.3732	0.1932	0.4395	0.5690	2.8215	1.8851	1.3729	2.0707
GRU	0.0062	0.0021	0.0458	0.1574	1.6608	1.0308	1.0152	1.0724

**Table 7:** Comparison of the proposed model (GRU) for HL and CL prediction with state-of-the-art models.

Method	HL			CL		
	MAE	MSE	RMSE	MAE	MSE	RMSE
Tsanas and Xifara [12]	0.51	-	-	1.42	-	-
Chou and Bui [22]	0.236	-	0.346	0.89	-	1.566
Cheng and Cao [23]	0.35	-	0.47	0.71	-	1
Sonmez et al. [25]	0.61	-	-	1.25	-	-
Alam et al. [26]	-	-	0.19	-	-	1.42
Regina and Capriles [28]	0.246	-	1.094	0.39	-	1.284
Nilashi et al. [31]	0.16	-	0.26	0.52	-	0.81
Nwulu [32]	0.977	-	1.228	1.654	-	2.111
Duarte et al. [33]	0.315	-	0.223	0.565	-	0.837
Roy et al. [2]	0.037	-	0.053	0.127	-	0.195
Kavaklioglu [34]	-	-	3.16	-	-	3.122
Kumar et al. [35]	0.138		0.321	0.134	-	0.646
Al-Rakhami et al. [36]	0.175	-	0.265	0.307	-	0.47
Sekhar et al. [4]	-	-	0.059		-	0.079
Sadeghi et al. [44]	0.2	-	0.263	0.485	-	0.69
Proposed (hold-out)	0.0102	0.0003	0.0166	0.0167	0.0006	0.0247
Proposed (10-fold)	0.0092	0.0001	0.0100	0.0021	0.0001	0.0100
